# Paeoniflorin shows chondroprotective effects under IL-1β stress by regulating circ-PREX1/miR-140-3p/WNT5B axis

**DOI:** 10.1186/s13018-023-04238-x

**Published:** 2023-10-10

**Authors:** Lan’e Wu, Runke Tang, Weibiao Xiong, Shuhua Song, Qian Guo, Qingqing Zhang

**Affiliations:** 1 Xiong Wei-biao Workroom, Jiangxi Province Hospital of Integrated Chinese and Western Medicine, Nanchang, 330003 Jiangxi People’s Republic of China; 2 Department of Rehabilitation, Jiangxi Province Hospital of Integrated Chinese and Western Medicine, No. 90, Bayi Road, Nanchang City, 330003 Jiangxi Province People’s Republic of China; 3 Department of Dermatology, Jiangxi Province Hospital of Integrated Chinese and Western Medicine, Nanchang, 330003 Jiangxi People’s Republic of China

**Keywords:** Paeoniflorin, KOA, Chondrocytes, Circ-PREX1, miR-140-3p, WNT5B

## Abstract

**Background:**

Osteoarthritis (OA) is a chronic and degenerative bone and joint disease, and paeoniflorin shows anti-arthritis role in OA. This study planned to investigate the mechanism related to chondroprotective role of paeoniflorin in OA.

**Methods:**

Real-time quantitative PCR and western blotting were performed to measure expression levels of circ-PREX1, microRNA (miR)-140-3p, Wingless-type MMTV integration site family, member 5B (WNT5B), B cell lymphoma (Bcl)-2, and Bcl-2 Associated X Protein (Bax). MTT assay, EdU assay, flow cytometry and enzyme-linked immunosorbent assay evaluated cell viability, proliferation, apoptosis and inflammatory response, respectively. Dual-luciferase reporter assay and RNA immunoprecipitation assay identified the relationship among circ-PREX1, miR-140-3p, and WNT5B.

**Results:**

IL-1β highly induced apoptosis rate, Bax expression and TNF-α product, accompanied with decreased cell viability, cell proliferation and IL-10 secretion, whereas these effects were partially reversed after paeoniflorin pretreatment. Expression of circ-PREX1 was upregulated and miR-140-3p was downregulated in cartilage tissues of patients with knee OA (KOA) and IL-1β-induced human chondrocytes (C28/I2). Circ-PREX1 overexpression and miR-140-3p silencing attenuated the suppressive effect of paeoniflorin in IL-1β-induced C28/I2 cells. Furthermore, miR-140-3p was negatively regulated by circ-PREX1. WNT5B was a downstream target of miR-140-3p and could be modulated by the circ-PREX1/miR-140-3p pathway in IL-1β-induced C28/I2 cells.

**Conclusion:**

Paeoniflorin might protect human chondrocytes from IL-1β-induced inflammatory injury via circ-PREX1-miR-140-3p-WNT5B pathway, suggesting a potential preventative agent and a novel target for the treatment of KOA.

**Supplementary Information:**

The online version contains supplementary material available at 10.1186/s13018-023-04238-x.

## Introduction

Knee osteoarthritis (KOA) is a chronic, progressive, and degenerative bone and joint disease in middle-aged and elderly populations [1], and ranks 11th among 291 diseases for disability globally [2]. Traditional Chinese medicines (TCM) have been accepted as a complementary therapy for KOA [3]. Paeoniflorin, a monoterpene glycoside [4], is one of pharmacologically active ingredients of several TCM used to treat OA lesions, such as Shu-Jing-Hwo-Shiee-Tang, Tougu Xiaotong capsule, and Shenjin Huoxue Mixture [5–7]. The anti-arthritis role of paeoniflorin has been reported [8], as well as in OA [9]. However, the underlying molecular mechanism of paeoniflorin in articular cartilage cells remains undisclosed till now.

The genetics and epigenetics continue to be a topic of substantial research in OA [10], and dozens of circular RNAs (circRNAs) studies have been published since 2016. The altered circRNA expression profiles have been analyzed in human OA cartilage, synovium and chondrocytes [11–13]. In terms of biological role, circRNAs are emerging essential regulators of apoptosis, autophagy inflammatory response, and extracellular matrix of chondrocytes [14, 15]. Moreover, circRNAs-microRNAs (miRNAs) regulation network is incorporated in the initiation and development of OA [12, 13, 16]. The circRNAs and miRNAs are also differently expressed in peripheral blood or serum of OA patients [17, 18], thus serving as potential diagnostic biomarkers. CircRNA hsa_circ_0060652, derived from PREX1 gene (referred as circ-PREX1) is highly expressed in knee joint chondrocytes of OA than that from Kashin-Beck disease [17]. However, the role of circ-PREX1 in OA chondrocytes has been unclear, as well as the related mechanism.

The objective of this study is to assess (1) circ-PREX1 and miR-140-3p contents in KOA patients and interleukin (IL)-1β-stimulated chondrocytes treated with paeoniflorin or not, and (2) effect and association of both genes in the protective role of paeoniflorin in IL-1β-induced human chondrocyte injury.

## Materials and methods

### Cartilage tissues

The KOA cartilage tissues were obtained from 30 patients with KOA (age 63.04 ± 3.56 years; 18 female, 12 male) during total knee joint replacement surgery. The control cartilage tissues were recruited from 30 traumatic patients (age 40.2 ± 6.2 years; 10 female, 20 male) undergoing amputation surgery. The control patients were free from OA or rheumatoid arthritis. These participators were from Jiangxi Province Hospital of Integrated Chinese and Western Medicine, and this study was pre-approved by the Institutional Review Board and Ethics Committee of Jiangxi Province Hospital of Integrated Chinese and Western Medicine. Human tissue collection was after received written informed consent from every patients.

### Cell culture

Human normal chondrocyte cells C28/I2 (BNCC339995; BNCC, Beijing, China) was cultured in DMEM/F-12 (GIBCO-BRL, Grand Island, NY, USA) containing 1% penicillin–streptomycin (GIBCO-BRL) at 37 °C with 5% CO_2_.

### Treatment of IL-1β and paeoniflorin

To mimic cell model of OA, C28/I2 cells were exposed to various concentrations (5, 10 or 20 ng/mL) of IL-1β (SRP3083; Sigma, St. Louis, MO, USA) for 24 h-incubation. For paeoniflorin treatment alone, C28/I2 cells were exposed to 25, 50 or 100 μM of paeoniflorin (P0038; Sigma-Aldrich) for 24 h. For co-treatment of IL-1β and paeoniflorin, C28/I2 cells were allowed to grow in complete medium added with paeoniflorin (50 μM) for 4 h, followed by IL-1β (10 ng/mL) treatment for another 24 h.

### RT-qPCR

RNA preparation was achieved through TRIpure (BioTeke, Beijing, China). The cDNA was synthesized by cDNA Synthesis Kit (Yeasen Biotech, Shanghai, China) for circ-PREX1 and WNT5B, and miScript Reverse Transcription kit (Yeasen Biotech) for miR-140-3p. The RNA expression was measured employing 5× All-In-One RT MasterMix (abm, Richmond, Vancouver, Canada) and special primer pairs on ABI7500 Real-time system. Primers of circ-PREX1 were 5′-ACGAAGGCCAAAGACGGATT-3′ and 5′-TCTTGGCCATGCAGACAAAC-3′, miR-140-3p were 5′-GCGCGTACCACAGGGTAGAA-3′ and 5′-AGTGCAGGGTCCGAGGTATT-3′, WNT5B were 5′-CAAGGAATGCCAGCACCAGTTC-3′ and 5′-CGGCTGATGGCGTTGACCACG-3′, GAPDH were 5′-GACAGTCAGCCGCATCTTCT-3′ and 5′-GCGCCCAATACGACCAAATC-3′, and U6 snoRNA (U6) were 5′-CGCGCTTCGGCAGCACATATACT-3′ and 5′-ACGCTTCACGAATTTGCGTGTC-3′. Target genes were quantified using the formula 2^−ΔΔCt^. GAPDH (for circ-PREX1 and WNT5B) and U6 (for miRNA) served as internal controls.

### Cell immunohistochemical staining

Type II collagen cell immunohistochemical staining was performed for cartilage cell activity identification. The cells were seeded on cover slides and grown into a monolayer. After removal, they were washed three times with PBS. Then, they were fixed with 4% paraformaldehyde and incubated with 3% hydrogen peroxide at room temperature. The cells were then blocked with 10% FBS medium, serum was discarded, and anti-type II collagen primary antibody (#ZRB1201, Sigma-Aldrich) was added and incubated overnight at 4 °C. Biotin-labeled goat anti-rabbit secondary antibody was added and incubated at room temperature, followed by three washes with PBS. DAB was used for visualization, followed by counterstaining with hematoxylin, and mounting with neutral quick-dry adhesive. Observation was conducted under a microscope.

### MTT assay

After treatment of IL-1β and/or paeoniflorin, MTT reagent (Sigma-Aldrich) was added in fresh medium without serum, and C28/I2 cells were incubated with MTT for another 4 h. Then, 150 μL of dimethyl sulfoxide was supplemented in each well followed by oscillation. Optical density (OD) value of soluble formazan in each well was detected using a microplate reader at the wavelength of 570 nm. Cell viability (%) = OD_experimental group_/OD_control group_ × 100%.

### EdU assay

C28/I2 cells grown in 48-well culture plates were used for proliferation analysis with EdU test reagents (Beyotime, Shanghai, China). DMEM/F-12 (GIBCO-BRL) was used to dilute EdU solution. After 2-h culture using the prepared EdU solution, paraformaldehyde and click reaction solution were used to incubate the samples. The stained cells by 4′,6-Diamidino-2-Phenylindole were photographed for proliferation analysis.

### Flow cytometry (FCM)

Apoptotic cells of chondrocyte cells pre-treated with paeoniflorin or not were subjected to flow cytometer analysis. The C28/I2 cells were added with FITC-labeled Annexin-V (Vazyme, Nanjing, China) and propidium iodide (Vazyme). Next, the stained cells were analyzed on accuri C6 flow cytometer (BD Biosciences, San Jose, CA, USA). Apoptosis rate (%) = the ratios of AnnexinV-FITC^+^/PI^+^  + AnnexinV-FITC^+^/PI^−^.

### Western blotting

The total protein was isolated using Radioimmunoprecipitation Assay (Pierce, Rockford, IL, USA) buffer. The protein samples were split, and 20 μg proteins were subjected to polyacrylamide gel electrophoresis and antibody incubation. The primary antibodies targeting Bcl-2 (sc-7382), Bax (sc-7480), WNT5B (sc-376249), and GAPDH (sc-32233) were purchased from Santa Cruz (Shanghai, China), as well as the secondary antibody targeting mouse IgG (sc-516102).

### ELISA

After IL-1β treatment, the cell culture supernate of C28/I2 cells was harvested, and the concentrations of TNF-α and IL-10 were measured using Human TNF-α Quantikine ELISA kit and Human IL-10 Quantikine ELISA kit, respectively. These kits were from R&D System (Minneapolis, MN, USA) and performed according to the product datasheets.

### Cell transfection

The pcDNA3.1/Hygro ( +) vector (pcDNA; Invitrogen, Carlsbad, CA, USA) was utilized to construct circ-PREX1-overexpression vector (pcDNA-circ-PREX1, circ-PREX1) and WNT5B-overexpression vector (pcDNA-WNT5B, WNT5B). The mimic of miR-140-3p (miR-140-3p mimic; 5′-UACCACAGGGUAGAACCACGG-3′), inhibitor of miR-140-3p (anti-miR-140-3p; 5′-CCGUGGUUCUACCCUGUGGUA-3′), and siRNA against circ-PREX1 (si-circ-PREX1; 5′-AAGGCCAAAGACGGATTACCA-3′) were chemically synthesized by Sangon Biotech (Shanghai, China), as well as the negative controls including miR-NC mimic (5′-UUUGUACUACACAAAAGUACUG-3′), anti-miR-NC (5′-UCUACUCUUUCUAGGAGGUUGGA-3′), and si-NC (5′-CCUAAGGUUAAGUCGCCCUCG-3′). For transfection, C28/I2 cells were transferred in 6-well plate and incubated with 40 nM of mimic, 20 nM of inhibitor or siRNA, and 2 μg of pcDNA vector using Lipofectamine RNAi MAX (Invitrogen). After 24 h-transfection, C28/I2 cells were collected for further treatment of IL-1β alone or co-treatment of paeoniflorin and IL-1β.

### Dual-luciferase reporter assay

Mutant types of circ-PREX1 (MUT-circ-PREX1) and MUT-WNT5B 3′UTR were acquired from Yeasen Biotech, thus the mutants showed no miR-140-3p binding sites. The wild types of circ-PREX1 (WT-circ-PREX1) and WT-WNT5B 3′UTR were separately inserted in pmiR-REPORT™ vectors (Promega, Madison, WI, USA), as well as the mutants. C28/I2 cells were transferred in 24-well plate and co-transfected with pmiR vectors (400 ng) and mimics (40 pmol) of miR-140-3p or miR-NC. After co-transfection for 48 h, luciferase activities were detected using Dual-Glo Luciferase Assay System (Promega).

### RNA immunoprecipitation (RIP)

The cell lysate of C28/I2 cells was collected, and subjected to RIP assay using Magna RIP RNA-Binding Protein Immunoprecipitation Kit (Millipore, Billerica, MA, USA). Antibodies targeting Ago2 (ab32381; Abcam, Cambridge, UK) and IgG (ab2410; Abcam) were pre-coupled with magnetic beads, respectively. The cell lysate was incubated with antibody-coated magnetic beads, and then digested by proteinase K. The extracted immunoprecipitated RNA was analyzed by RT-qPCR.

### Statistical analysis

The results were displayed as mean ± standard deviation. The data analysis was performed on GraphPad Prism 7.0. Comparisons were performed with Student’s *t*-test or one-way analysis of variance followed with Turkey’s post hoc test. The data were deemed to be statistically significant at *P* < 0.05.

## Results

### Circ-PREX1 and miR-140-3p were abnormally expressed in KOA cartilage tissues and IL-1β-induced human chondrocytes

**Fig. 1:**
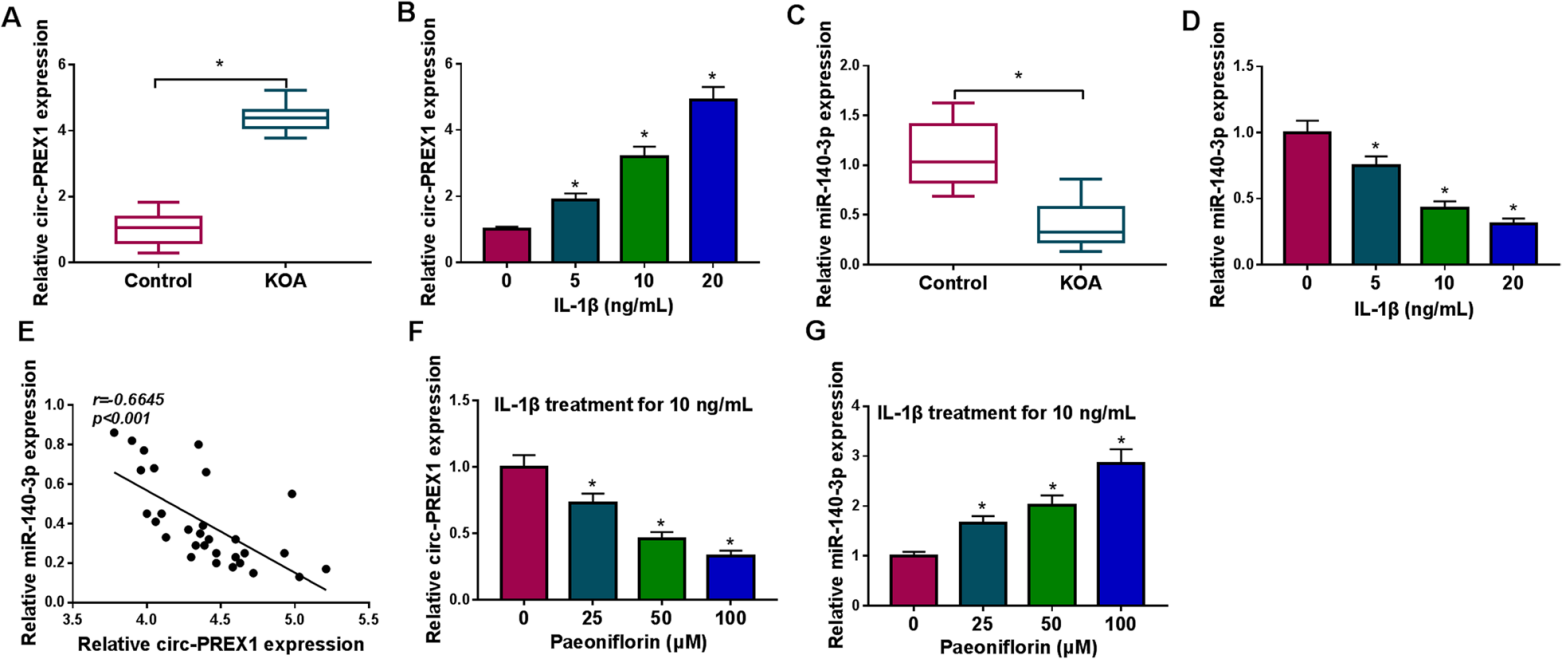
Expression of hsa_circ_0060652 (circ-PREX1) and miRNA (miR)-140-3p in knee osteoarthritis (KOA) patients and interleukin (IL)-1β-induced human chondrocytes. **A-D** RT-qPCR measured circ-PREX1 and miR-140-3p levels in (**A**, **C**) KOA cartilage tissues (KOA; n = 30) and control cartilage tissues (Control; n = 30), and **B**, **D** human normal chondrocyte cell line (C28/I2) treated with IL-1β (0, 5, 10 and 20 ng/mL) for 24 h. **E** Pearson’s correlation (*r*) analysis analyzed the correlation between circ-PREX1 and miR-140-3p in KOA cartilages (n = 30). **F**, **G** RT-qPCR measured circ-PREX1 and miR-140-3p levels in IL-1β-induced C28/I2 cells pre-treated with paeoniflorin (0, 25, 50 and 100 μM) for 4 h. *P < 0.05

The KOA cartilages were isolated from knee joints of patients with KOA, and expression of circ-PREX1 and miR-140-3p was further measured. As RT-qPCR analysis showed, circ-PREX1 level was 4.2-fold and miR-140-3p was 0.3-fold of that in control cartilages (Fig. 1A and C). The synthesis and secretion of type II collagen are important characteristics for maintaining the differentiated phenotype of chondrocytes. Identification of C28/I2 cells was performed through type II collagen staining, with most of the cellular staining located in the cytoplasm and appearing as a deep brown color (Additional file 1: Figure S1). Besides, circ-PREX1 was gradually upregulated in C28/I2 cells treated with 5–20 ng/mL of IL-1β, accompanied with downregulated miR-140-3p (Fig. 1B and D). Furthermore, there was an inverse correlation between circ-PREX1 and miR-140-3p in KOA cartilages (Fig. 1E). These data might suggest a reciprocal action of circ-PREX1 and miR-140-3p in the development of KOA.

### Paeoniflorin mitigated IL-1β-induced apoptosis and inflammatory response in human chondrocytes in vitro

**Fig. 2:**
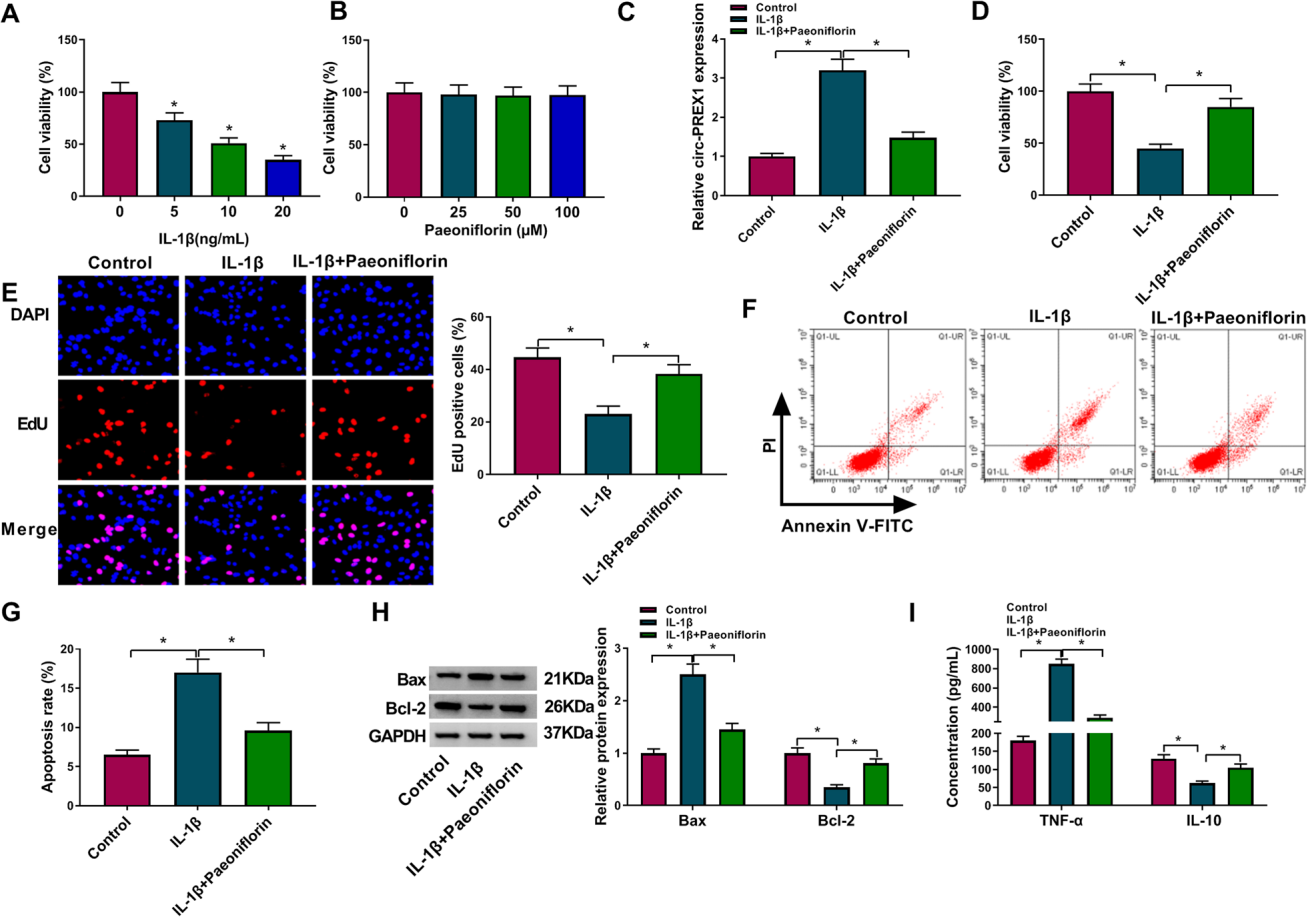
The role of paeoniflorin in IL-1β-induced human chondrocytes in vitro. **A**, **B** MTT assay assessed cell viability of C28/I2 cells treated with IL-1β (0, 5, 10 and 20 ng/mL) for 24 h or paeoniflorin (0, 25, 50 and 100 μM) for 24 h. **C-I** IL-1β-induced C28/I2 cells were pre-treated with paeoniflorin (50 μM) for 4 h or not, and **C** RT-qPCR detected circ-PREX1 level, **D** MTT assay assessed cell viability, **E** EdU assay was performed to analyze cell proliferation, **F** and **G** flow cytometry (FCM) examined apoptosis rate, **H** western blotting measured protein levels of Bcl-2 and Bax, and **I** ELISA determined products of TNF-α and IL-10. *P < 0.05

Recently, paeoniflorin had been identified to exert anti-arthritic effects. Expression of circ-PREX1 was decreased, and miR-140-3p was increased with pretreatment of paeoniflorin in IL-1β-induced C28/I2 cells (Fig. 1F and G). MTT assay showed that IL-1β stimulated cell viability inhibition in C28/I2 cells (Fig. 2A), while paeoniflorin displayed little effect on that (Fig. 2B). Besides, 50 μM of paeoniflorin attenuated circ-PREX1 expression level in IL-1β-induced C28/I2 cells (Fig. 2C). With paeoniflorin pre-treatment, IL-1β-induced inhibition in cell viability and proliferation was rescued in C28/I2 cells (Fig. 2D and E). The high apoptosis rate of IL-1β-induced C28/I2 cells was reduced due to paeoniflorin pretreatment (Fig. 2F and G), accompanied with elevated Bcl-2 and promoted Bax (Fig. 2H). Paeoniflorin also suppressed TNF-α (pro-inflammatory cytokine [19]) secretion and improved IL-10 (anti-inflammatory cytokine [19]) secretion in C28/I2 cells under IL-1β stress (Fig. 2I). These results demonstrated that paeoniflorin mitigated IL-1β-induced apoptosis and inflammatory response in human chondrocytes in vitro accompanying with circ-PREX1 downregulation.

### Overexpression of circ-PREX1 and downregulation of miR-140-3p attenuated the protective role of paeoniflorin in IL-1β-induced human chondrocytes in vitro

**Fig. 3:**
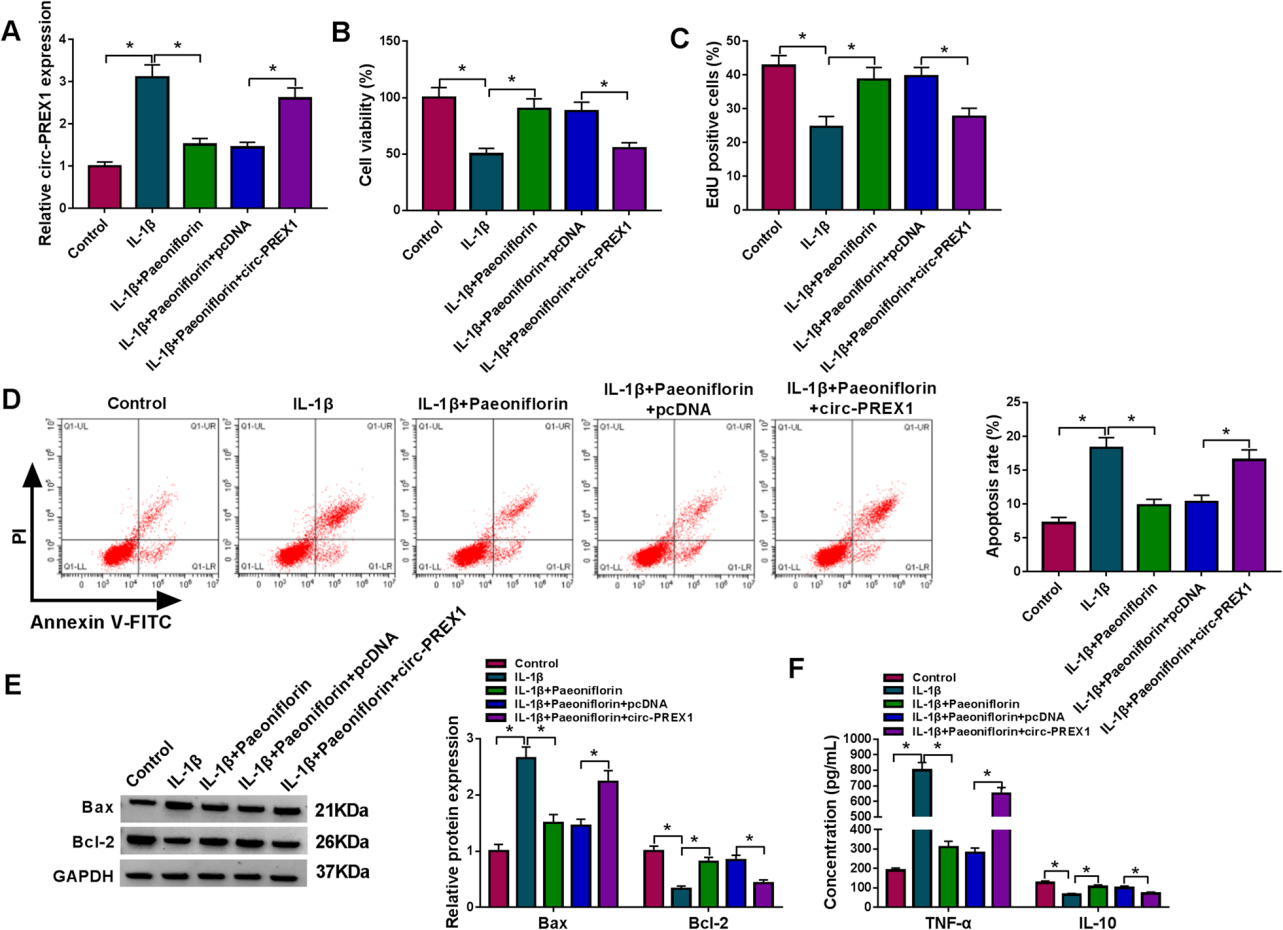
Effect of circ-PREX1 on the protective role of paeoniflorin in IL-1β-induced human chondrocytes in vitro. IL-1β-induced C28/I2 cells were pre-transfected with pcDNA-circ-PREX1 (circ-PREX1) or the empty vector (pcDNA) and then treated with paeoniflorin (50 μM) for 4 h. **A** RT-qPCR detected circ-PREX1 level, **B** MTT assay assessed cell viability, **C** EdU assay was performed to analyze cell proliferation, **D** FCM examined apoptosis rate, **E** western blotting measured protein levels of Bcl-2 and Bax, and **F** ELISA determined products of TNF-α and IL-10. *P < 0.05

**Fig. 4:**
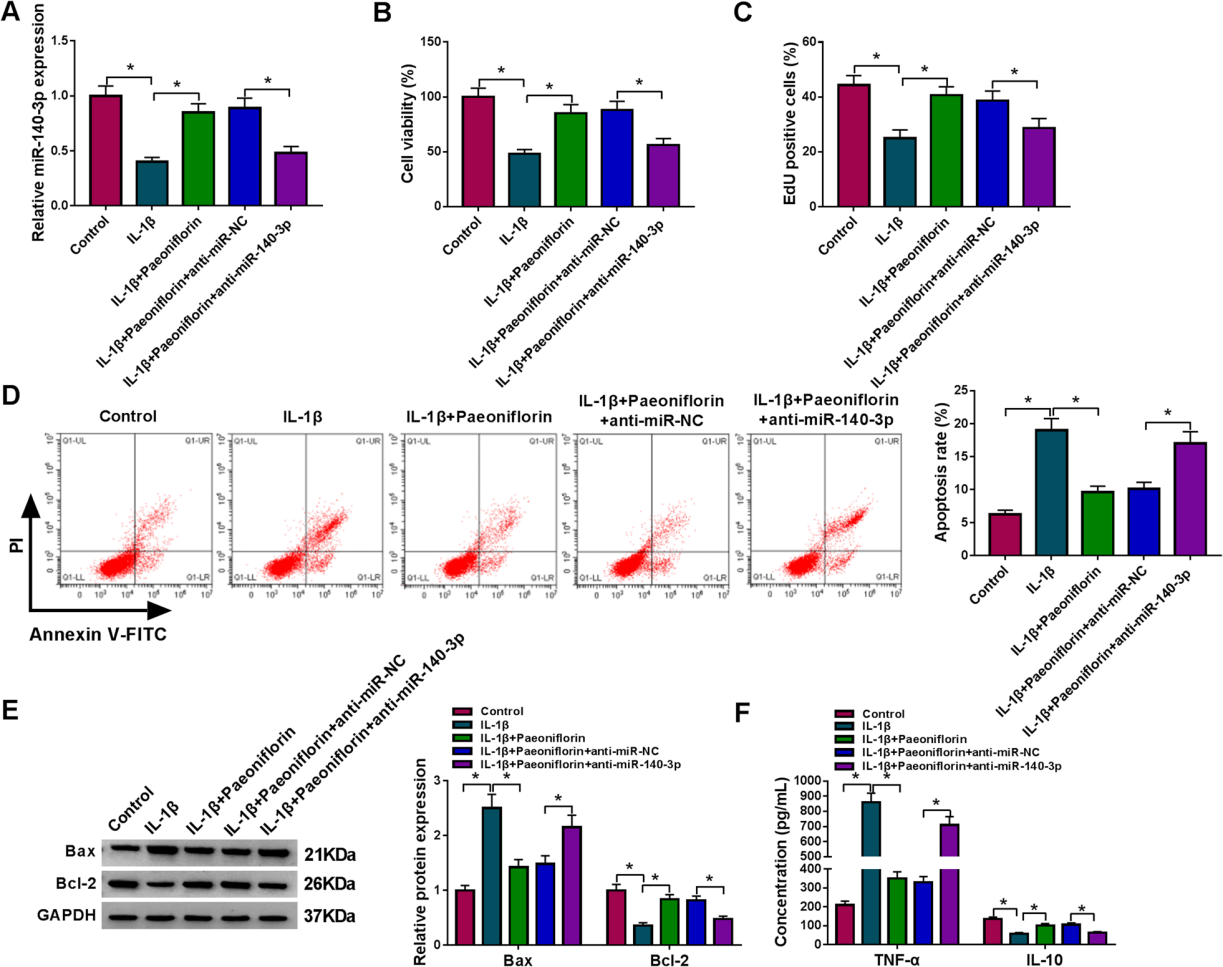
Effect of miR-140-3p on the protective role of paeoniflorin in IL-1β-induced human chondrocytes in vitro. IL-1β-induced C28/I2 cells were pre-transfected with miR-140-3p inhibitor (anti-miR-140-3p) or the negative control (anti-miR-NC) and then treated with paeoniflorin (50 μM) for 4 h. **A** RT-qPCR detected miR-140-3p level, **B** MTT assay assessed cell viability, **C** EdU assay was performed to analyze cell proliferation, **D** FCM examined apoptosis rate, **E** western blotting measured protein levels of Bcl-2 and Bax, and **F** ELISA determined products of TNF-α and IL-10. *P < 0.05

The contribution of circ-PREX1 to the protective role of paeoniflorin was further identified in IL-1β-induced inflammatory injury in human chondrocytes. C28/I2 cells were transfected with circ-PREX1 vector or pcDNA empty vector, and followed with co-treatment of paeoniflorin and IL-1β. The high efficiency of circ-PREX1 overexpression was shown in Additional file 2: Figure S2A. In IL-1β-induced C28/I2 cells pre-treated with paeoniflorin, expression of circ-PREX1 was inhibited, and circ-PREX1 vector then recovered circ-PREX1 expression (Fig. 3A). Circ-PREX1 recovery inhibited cell viability and proliferation and induced cell apoptosis, as evidenced by lowered cell viability, the number of EdU-positive cells and Bcl-2 expression (Fig. 3B, C and E), and elevated apoptosis rate and Bax expression (Fig. 3D and E). The high level of IL-10 and low level of TNF-α in IL-1β-induced C28/I2 cells pre-treated with paeoniflorin were reversed by circ-PREX1 upregulation (Fig. 3F). These outcomes suggested a suppressive effect of circ-PREX1 overexpression in the protective role of paeoniflorin in IL-1β-induced inflammatory injury in human chondrocytes in vitro. As shown in Additional file 2: Figure S2B, miR-140-3p inhibitor was effective in decreasing miR-140-3p expression. Anti-miR-140-3p transfection mediated miR-140-3p knockdown in IL-1β-induced C28/I2 cells pre-treated with paeoniflorin (Fig. 4A), and this action led to a decrease of cell viability, cell proliferation, Bcl-2 expression and IL-10 secretion (Fig. 4B, C, E and F), as well as an increase of apoptosis rate, Bax expression and TNF-α secretion (Fig. 4D–F). Collectively, upregulating circ-PREX1 or downregulating miR-140-3p could diminish the protective role of paeoniflorin in IL-1β-induced inflammatory injury in human chondrocytes in vitro.

### There was a target relationship between circ-PREX1 and miR-140-3p

**Fig. 5:**
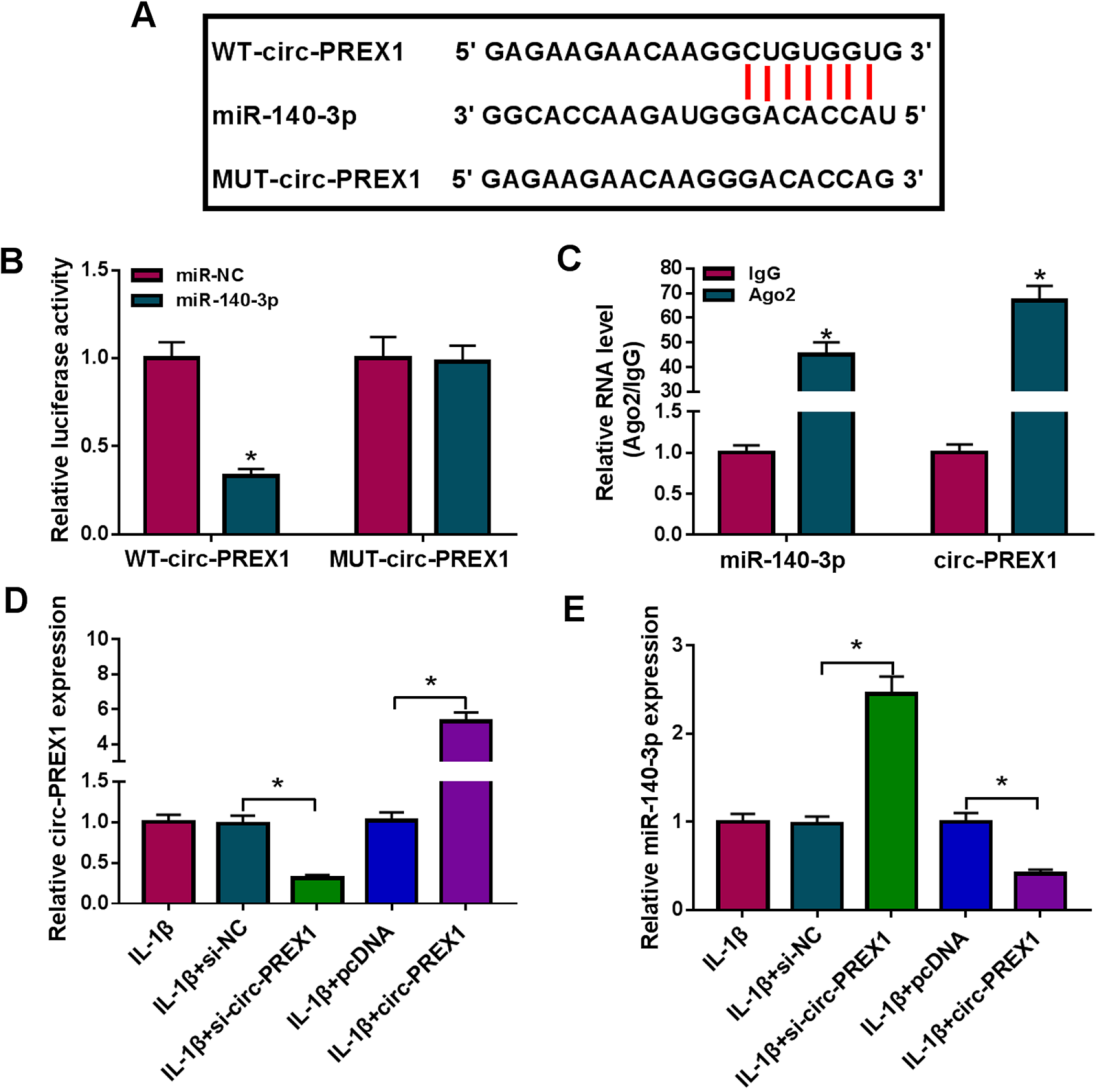
The relationship between circ-PREX1 and miR-140-3p. **A** The diagram showed the putative binding sites among miR-140-3p, the wild type of circ-PREX1 (WT-circ-PREX1) and its mutant (MUT-circ-PREX1). **B** Dual-luciferase reporter assay measured luciferase activity of report vectors carrying WT-circ-PREX1 or MUT-circ-PREX1 in C28/I2 cells transfected with miR-140-3p mimic (miR-140-3p) or its negative control (miR-NC). **C** RNA immunoprecipitation (RIP) determined the enrichment levels of circ-PREX1 and miR-140-3p in C28/I2 cells. **D**, **E** RT-qPCR detected circ-PREX1 and miR-140-3p levels in IL-1β-induced C28/I2 cells pre-transfected with pcDNA, circ-PREX1, or siRNA against circ-PREX1 or scrambled RNA (si-circ-PREX1 or si-NC). *P < 0.05

Moreover, circinteractome software (https://circinteractome.nia.nih.gov/) predicted a complementary relationship between circ-PREX1 and miR-140-3p, and the putative binding sites were presented in Fig. 5A. The high efficiency of miR-140-3p mimic in upregulating miR-140-3p expression was confirmed by RT-qPCR (Additional file 2: Figure S2B). Using dual-luciferase reporter assay, luciferase activity of pmiR vectors carrying WT-circ-PREX1 was attenuated by miR-140-3p mimic transfection in C28/I2 cells (Fig. 5B); additionally, RIP assay depicted a simultaneously remarkable enrichment of circ-PREX1 and miR-140-3p by Ago2 in C28/I2 cells (Fig. 5C). Subsequently, the data of RT-qPCR revealed that siRNA of circ-PREX1 was effective in downregulating circ-PREX1 expression in IL-1β-treated C28/I2 cells (Additional file 2: Figure S2A). The expression level of miR-140-3p was downregulated by circ-PREX1 overexpression via pcDNA vector transfection, and upregulated by circ-PREX1 knockdown via siRNA transfection in IL-1β-induced C28/I2 cells (Fig. 5D and E). These data could indicate a direct regulatory effect of circ-PREX1 on miR-140-3p in human chondrocytes.

### WNT5B was a downstream target of miR-140-3p

**Fig. 6:**
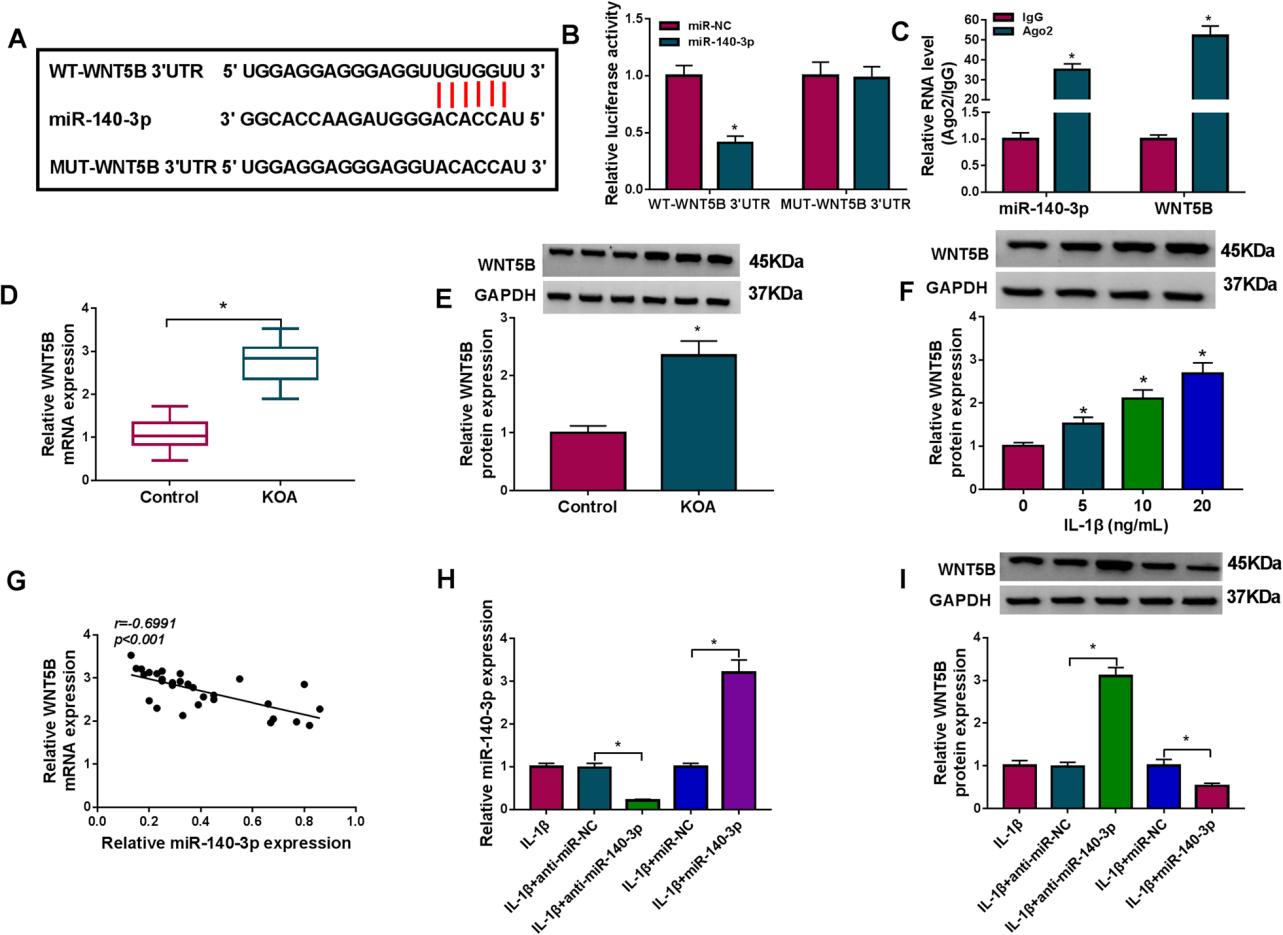
The identification of target of miR-140-3p. **A** The diagram showed the putative binding sites among miR-140, the wild type of WNT5B 3′UTR (WT-WNT5B 3′UTR) and its mutant (MUT-WNT5B 3′UTR). **B** Dual-luciferase reporter assay measured luciferase activity of report vector carrying WT-WNT5B 3′UTR or MUT-WNT5B 3′UTR in C28/I2 cells transfected with miR-140-3p or miR-NC. **C** RIP determined the enrichment levels of WNT5B and miR-140-3p in C28/I2 cells. **D–F** RT-qPCR and western blotting measured WNT5B mRNA level and protein level in (**D**, **E**) tissues in KOA group (n = 30) and Control group (n = 30), and **F** C28/I2 cells treated with IL-1β (0, 5, 10 and 20 ng/mL) for 24 h. **G** Pearson’s correlation (*r*) analysis analyzed the correlation betweenWNT5B mRNA and miR-140-3p in KOA cartilages (n = 30). **H**, **I** RT-qPCR and western blotting detected miR-140-3p and WNT5B levels in IL-1β-induced C28/I2 cells pre-transfected with anti-miR-NC, anti-miR-140-3p, miR-NC, or miR-140-3p. *P < 0.05

The starBase v2.0 software (http://starbase/miRNA&mRNAs/==hsa-miR-140-3p&clipNum/=wnt5b) also provided a potent interaction between miR-140-3p and WNT5B (Fig. 6A). With the mutation of the core sites, miR-140-3p mimic transfection failed to alter the luciferase activity of pmiR vector carrying MUT-WNT5B 3′UTR (Fig. 6B). MiR-140-3p and WNT5B were simultaneously enriched by Ago2 in C28/I2 cells (Fig. 6C). These results indicated WNT5B was a target gene of miR-140-3p. Then, expression of WNT5B in KOA was investigated. The mRNA expression of WNT5B was significantly upregulated in human KOA cartilage tissues (Fig. 6D), as well as its protein level (Fig. 6E). Similarly, WNT5B protein level was gradually increased in C28/I2 cells stimulated by different concentrations of IL-1β (Fig. 6F). There was a negative correlation between miR-140-3p and WNT5B expression in this group of KOA patients (Fig. 6G). Besides, expression levels of WNT5B were downregulated by miR-140-3p overexpression via mimic transfection, and upregulated by miR-140-3p knockdown via inhibitor transfection in IL-1β-induced C28/I2 cells (Fig. 6H and I). These outcomes showed a direct inhibitory regulation of miR-140-3p on WNT5B in human chondrocytes.

### Paeoniflorin modulated circ-PREX1/miR-140-3p/WNT5B axis in IL-1β-induced human chondrocytes in vitro

**Fig. 7:**
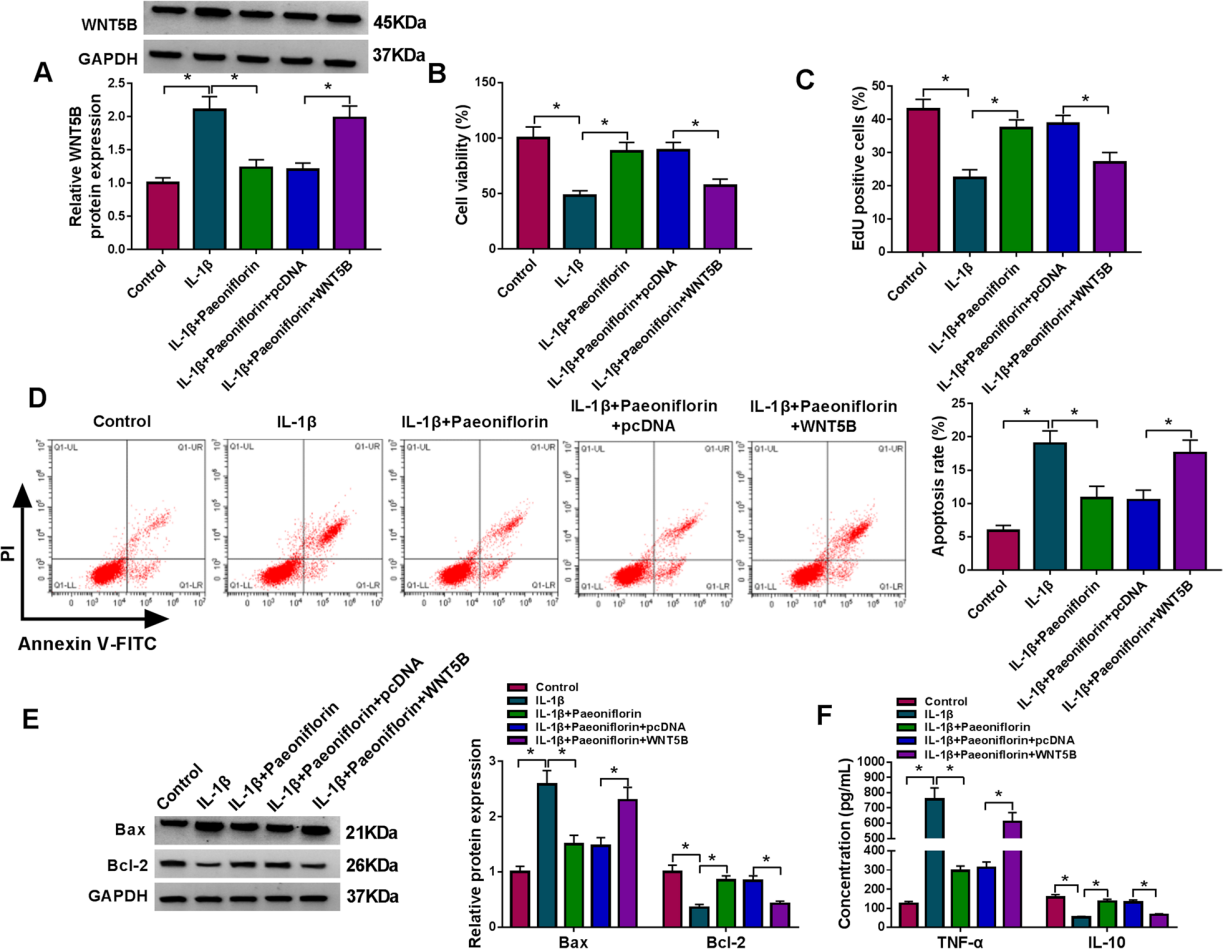
Effect of WNT5B on the protective role of paeoniflorin in IL-1β-induced human chondrocytes in vitro. IL-1β-induced C28/I2 cells were pre-transfected with pcDNA-WNT5B (WNT5B) or pcDNA prior to paeoniflorin (50 μM) treatment for 4 h. **A** Western blotting detected WNT5B protein expression level. **B** MTT assay assessed cell viability. **C** EdU assay was performed to analyze cell proliferation. **D** FCM examined apoptosis rate. **E** Western blotting measured protein levels of Bcl-2 and Bax. **F** ELISA determined products of TNF-α and IL-10. *P < 0.05

**Fig. 8:**
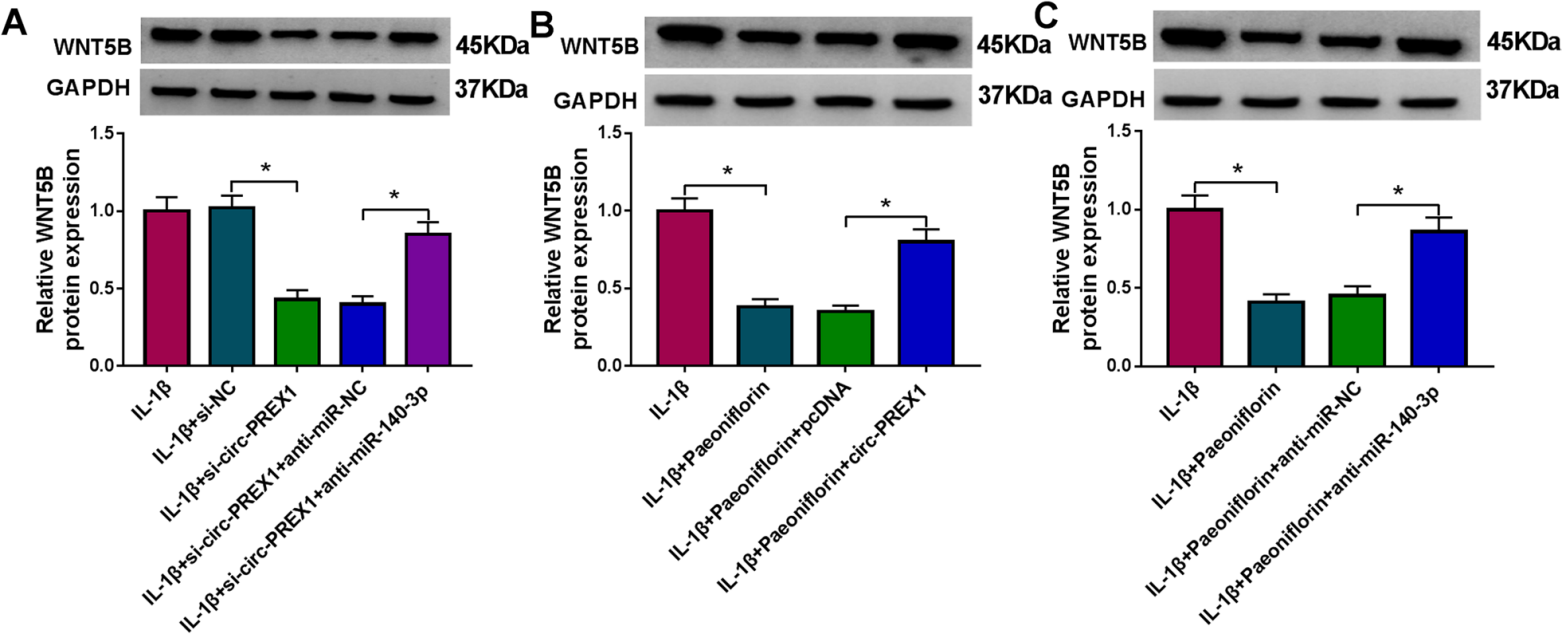
Paeoniflorin modulated circ-PREX1/miR-140-3p/WNT5B axis in IL-1β-induced human chondrocytes in vitro. **A** Western blotting examined WNT5B protein level in IL-1β-induced C28/I2 cells pre-transfected with si-NC, si-circ-PREX1, si-circ-PREX1 and anti-miR-NC, or si-circ-PREX1 and anti-miR-140-3p. **B**, **C** Western blotting examined WNT5B protein level in IL-1β-induced C28/I2 cells pre-transfected with circ-PREX1, pcDNA, anti-miR-140-3p, or anti-miR-NC, and then treated with paeoniflorin (50 μM) for 4 h. *P < 0.05

As shown in Additional file 2: Figure S2C, WNT5B overexpression plasmid was effective in upregulating WNT5B expression in IL-1β-induced C28/I2 cells. Re-expression of WNT5B could also counteract the effects of paeoniflorin on WNT5B protein expression, cell viability, cell proliferation, apoptosis rate, and expression levels of Bcl-2, Bax, IL-10, and TNF-α in IL-1β-induced C28/I2 cells (Fig. 7A–F). Moreover, expression of WNT5B protein was also suppressed by circ-PREX1 deficiency via siRNA transfection, and this suppression could be relieved by presence of anti-miR-140-3p in IL-1β-induced C28/I2 cells (Fig. 8A). Besides, the high expression of WNT5B protein in IL-1β-induced C28/I2 cells was declined with paeoniflorin pretreatment, which was rescued by both circ-PREX1 overexpression and miR-140-3p silencing (Fig. 8B and C). These results suggested that paeoniflorin could modulate the circ-PREX1/miR-140-3p/WNT5B axis to participate in IL-1β-induced human chondrocytes in vitro.

## Discussion

IL-1β, an early-stage inflammatory cytokine, had been commonly used to induce an in vitro OA model [20], and IL-1β was highly potent to induce proliferation, differentiation, apoptosis, inflammation, and ECM synthesis of OA chondrocytes [21]. Paeoniflorin was considered to exert anti-inflammation, anti-apoptosis and anti-fibrosis roles in model of OA in chondrocytes induced by IL-1β [9, 22, 23]. Here, we attempted to explore the informative RNAs underlying the protective role of paeoniflorin in IL-1β-induced chondrocytes.

With paeoniflorin pretreatment, apoptosis rate, Bax expression and TNF-α secretion were decreased, meanwhile cell viability, cell proliferation, Bcl-2 expression and IL-10 secretion were improved in the OA cell model. These data suggested that paeoniflorin displayed inhibitory effects on IL-1β-induced chondrocyte apoptosis as well as inflammation and promoting effect on chondrocyte proliferation under IL-1β treatment in vitro, which was similar to previous studies [9, 22, 23]. More importantly, circ-PREX1 was highly induced in the OA cell model, which was downregulated by paeoniflorin during its protective role in apoptosis and inflammatory response. This might be the first evidence of the relationship between paeoniflorin and circ-PREX1. By the way, paeoniflorin functioned in diverse cancers and inflammatory disorders [4, 24], it would be great helpful to determine the reciprocal interaction between paeoniflorin and circ-PREX1 in these diseases. Besides, circ-PREX1, one of the top 10 most upregulated circRNAs, was upregulated to 5.73-fold in human KOA chondrocytes versus patients with Kashin-Beck disease [17]. Therefore, circ-PREX1 might be an OA-related circRNA; and target miRNAs of circ-PREX1 should also be further identified. Thus, in this study, we searched and identified miR-140-3p as a target of circ-PREX1 according to circinteractome software, luciferase assay and RIP assay.

A previous study revealed that miR-140-3p was the most highly expressed miRNA in healthy cartilage, and increased during in vitro chondrogenesis [25]. We observed a downregulation of miR-140-3p in human KOA cartilage tissues and IL-1β-induced chondrocytes, which was consistent with preceding data [26, 27]. Moreover, it was discovered to be downregulated in synovial fluid of OA patients, as well [18, 28, 29]. Furthermore, miR-140-3p together with several other miRNAs were documented to be biomarkers to evaluate OA severity and progression [18, 28, 29]. We noticed that miR-140-3p was elevated due to paeoniflorin pretreatment, and silencing miR-140-3p could diminish the effect of paeoniflorin on cell viability, cell proliferation, apoptosis rate, expression of Bcl-2 and Bax, as well as secretions of TNF-α and IL-10 in IL-1β-induced chondrocytes in vitro. Among these outcomes, the anti-apoptosis role of miR-140-3p overexpression in IL-1β-induced chondrocytes had also been preliminary annotated by Ren et al. [26]. In addition, miR-140-3p deletion was previously uncovered to exert suppressive activity on inflammatory response of chondrocytes associated with OA [27]. However, this study indicated a novel relationship between paeoniflorin and miR-140-3p. By the way, ECM-related proteins such as collagen II, aggrecan and matrix metalloproteinases were modulated by miR-140-3p dysregulation [26]; whereas, this performance was not further explored in the protective activities of paeoniflorin in this study, as well as autophagy, oxidative stress and cell metabolism [25].

WNT5B was one of WNT ligands that highly expressed in OA joint [30]. It was reported that WNT5B was important for chondrocyte differentiation and organization [30, 31]. WNT5B via exosome enhanced chondrocyte proliferation and migration to prevent the development of KOA [32]. Here, we noticed that WNT5B was a novel target gene of miR-140-3p in OA chondrocytes, except for ADAMTS5 and CXCR4 [26, 27]. Functionally, WNT5B lower expression was correlated with the protective trait of paeoniflorin in IL-1β-induced chondrocytes with regulation by circ-PREX1-miR-140-3p axis. By the way, silencing of WNT5B could prevent human chondrocytes CHON-001 from LPS-induced apoptosis by being targeted by miR-374a-3p [33].

Even though underlying signaling pathways were undefined, we demonstrated that overexpression of circ-PREX1 and/or silencing of miR-140-3p suppressed the protective effect of paeoniflorin on IL-1β-induced apoptosis and inflammatory response in chondrocytes in vitro by regulating WNT5B*.* Thus, this study suggested that circ-PREX1-miR-140-3p-WNT5B axis might be a novel molecular regulation mechanism of paeoniflorin exerting anti-arthritis role in KOA chondrocytes.

### Supplementary Information

**Additional file 1: Fig. S1.** Identification of C28/I2 cells was performed through type II collagen staining assay.

**Additional file 2: Fig. S2.** Analysis of circ-PREX1, miR-140-3p and WNT5B expression. (A) The effects of siRNA of circ-PREX1 and circ-PREX1 overexpression plasmid on circ-PREX1 expression were analyzed by RT-qPCR. (B) The effects of miR-140-3p mimic and inhibitor on miR-140-3p expression were determined by RT-qPCR. (C) The effect of WNT5B overexpression plasmid on WNT5B expression was determined by western blotting. *P < 0.05.

## Data Availability

The analyzed data sets generated during the present study are available from the corresponding author on reasonable request.

## Abbreviations

OA: Osteoarthritis
KOA: Knee OA
TCM: Traditional Chinese medicines
GAPDH: Glyceraldehyde-phosphate dehydrogenase
Ct: Cycle threshold
OD: Optical density
FITC: Fluorescein isothiocyanate
RIP: RNA immunoprecipitation

## References

[1] Felson DT, Lawrence RC, Dieppe PA, Hirsch R, Helmick CG, Jordan JM (2000). Osteoarthritis: new insights. Part 1: the disease and its risk factors. Ann Intern Med.

[2] Disease GBD, Injury I, Prevalence C (2016). Global, regional, and national incidence, prevalence, and years lived with disability for 310 diseases and injuries, 1990–2015: a systematic analysis for the Global Burden of Disease Study 2015. Lancet.

[3] Yang M, Jiang L, Wang Q, Chen H, Xu G (2017). Traditional Chinese medicine for knee osteoarthritis: an overview of systematic review. PLoS ONE.

[4] Xiang Y, Zhang Q, Wei S, Huang C, Li Z, Gao Y (2020). Paeoniflorin: a monoterpene glycoside from plants of Paeoniaceae family with diverse anticancer activities. J Pharm Pharmacol.

[5] Ueng YF, Lu CK, Yang SH, Wang HJ, Huang CC (2017). Potentiation of the anticoagulation effect of warfarin by the herbal remedy Shu-Jing-Hwo-Shiee-Tang in rats: the dosing regimen and pharmacokinetic interaction. Drug Metab Pharmacokinet.

[6] Li X, Zhang Z, Liang W, Zeng J, Shao X, Xu L (2020). Data on Tougu Xiaotong capsules may inhibit p38 MAPK pathway-mediated inflammation in vitro. Data Brief.

[7] Yu MX, Ma XQ, Song X, Huang YM, Jiang HT, Wang J (2020). Validation of the key active ingredients and anti-inflammatory and analgesic effects of Shenjin Huoxue mixture against osteoarthritis by integrating network pharmacology approach and thin-layer chromatography analysis. Drug Des Dev Ther.

[8] Zheng YQ, Wei W, Zhu L, Liu JX (2007). Effects and mechanisms of Paeoniflorin, a bioactive glucoside from paeony root, on adjuvant arthritis in rats. Inflamm Res.

[9] Zhao L, Chang Q, Huang T, Huang C (2018). Paeoniflorin inhibits IL1betainduced expression of inflammatory mediators in human osteoarthritic chondrocyte. Mol Med Rep.

[10] Jeffries MA (2019). Osteoarthritis year in review 2018: genetics and epigenetics. Osteoarthritis Cartilage.

[11] Li H, Yang HH, Sun ZG, Tang HB, Min JK (2019). Whole-transcriptome sequencing of knee joint cartilage from osteoarthritis patients. Bone Joint Res.

[12] Xiang S, Li Z, Bian Y, Weng X (2019). RNA sequencing reveals the circular RNA expression profiles of osteoarthritic synovium. J Cell Biochem.

[13] Wang Y, Wu C, Zhang F, Zhang Y, Ren Z, Lammi MJ (2019). Screening for differentially expressed circular RNAs in the cartilage of osteoarthritis patients for their diagnostic value. Genet Test Mol Biomark.

[14] Jiang S, Liu Y, Xu B, Zhang Y, Yang M. Noncoding RNAs: New regulatory code in chondrocyte apoptosis and autophagy. Wiley interdisciplinary reviews RNA. 2020:e1584. 10.1002/wrna.1584 31925936

[15] Wu Y, Lu X, Shen B, Zeng Y (2019). The therapeutic potential and role of miRNA, lncRNA, and circRNA in osteoarthritis. Curr Gene Ther.

[16] Kulcheski FR, Christoff AP, Margis R (2016). Circular RNAs are miRNA sponges and can be used as a new class of biomarker. J Biotechnol.

[17] Wang Y, Wu C, Zhang Y, Yang Y, Ren Z, Lammi MJ (2020). Screening for differentially expressed circRNA between Kashin-Beck disease and osteoarthritis patients based on circRNA chips. Clin Chim Acta.

[18] Ntoumou E, Tzetis M, Braoudaki M, Lambrou G, Poulou M, Malizos K (2017). Serum microRNA array analysis identifies miR-140-3p, miR-33b-3p and miR-671-3p as potential osteoarthritis biomarkers involved in metabolic processes. Clin Epigenetics.

[19] Wojdasiewicz P, Poniatowski LA, Szukiewicz D (2014). The role of inflammatory and anti-inflammatory cytokines in the pathogenesis of osteoarthritis. Mediators Inflamm.

[20] Chow YY, Chin KY (2020). The role of inflammation in the pathogenesis of osteoarthritis. Mediators Inflamm.

[21] Jenei-Lanzl Z, Meurer A, Zaucke F (2019). Interleukin-1beta signaling in osteoarthritis - chondrocytes in focus. Cell Signal.

[22] Lu M, Wang Y, Zhou S, Xu J, Li J, Tao R (2018). MicroRNA-370 suppresses the progression and proliferation of human astrocytoma and glioblastoma by negatively regulating beta-catenin and causing activation of FOXO3a. Exp Ther Med.

[23] Hu PF, Sun FF, Jiang LF, Bao JP, Wu LD (2018). Paeoniflorin inhibits IL-1beta-induced MMP secretion via the NF-kappaB pathway in chondrocytes. Exp Ther Med.

[24] Xin Q, Yuan R, Shi W, Zhu Z, Wang Y, Cong W (2019). A review for the anti-inflammatory effects of paeoniflorin in inflammatory disorders. Life Sci.

[25] Al-Modawi RN, Brinchmann JE, Karlsen TA (2019). Multi-pathway protective effects of MicroRNAs on human chondrocytes in an in vitro model of osteoarthritis. Mol Ther Nucleic Acids.

[26] Ren T, Wei P, Song Q, Ye Z, Wang Y, Huang L (2020). MiR-140-3p ameliorates the progression of osteoarthritis via targeting CXCR4. Biol Pharm Bull.

[27] Rasheed Z, Rasheed N, Al-Shaya O (2018). Epigallocatechin-3-O-gallate modulates global microRNA expression in interleukin-1beta-stimulated human osteoarthritis chondrocytes: potential role of EGCG on negative co-regulation of microRNA-140-3p and ADAMTS5. Eur J Nutr.

[28] Yin CM, Suen WC, Lin S, Wu XM, Li G, Pan XH (2017). Dysregulation of both miR-140-3p and miR-140-5p in synovial fluid correlate with osteoarthritis severity. Bone Joint Res.

[29] Kwak YH, Kwak DK, Kim NY, Kim YJ, Lim JS, Yoo JH (2020). Significant changes in synovial fluid microRNAs after high tibial osteotomy in medial compartmental knee osteoarthritis: identification of potential prognostic biomarkers. PLoS ONE.

[30] Huang J, Chen C, Liang C, Luo P, Xia G, Zhang L (2020). Dysregulation of the Wnt Signaling pathway and synovial stem cell dysfunction in osteoarthritis development. Stem Cells Dev.

[31] Sisson BE, Dale RM, Mui SR, Topczewska JM, Topczewski J (2015). A role of glypican4 and wnt5b in chondrocyte stacking underlying craniofacial cartilage morphogenesis. Mech Dev.

[32] Tao SC, Yuan T, Zhang YL, Yin WJ, Guo SC, Zhang CQ (2017). Exosomes derived from miR-140-5p-overexpressing human synovial mesenchymal stem cells enhance cartilage tissue regeneration and prevent osteoarthritis of the knee in a rat model. Theranostics.

[33] Shi FL, Ren LX (2020). Up-regulated miR-374a-3p relieves lipopolysaccharides induced injury in CHON-001 cells via regulating Wingless-type MMTV integration site family member 5B. Mol Cell Probes.

