# Supplementary figures and images for: Paeoniflorin shows chondroprotective effects under IL-1β stress by regulating circ-PREX1/miR-140-3p/WNT5B axis
Source: J Orthop Surg Res. 2023 Oct 10;18:766. doi: 10.1186/s13018-023-04238-x (PMC10566156; doi:10.1186/s13018-023-04238-x)

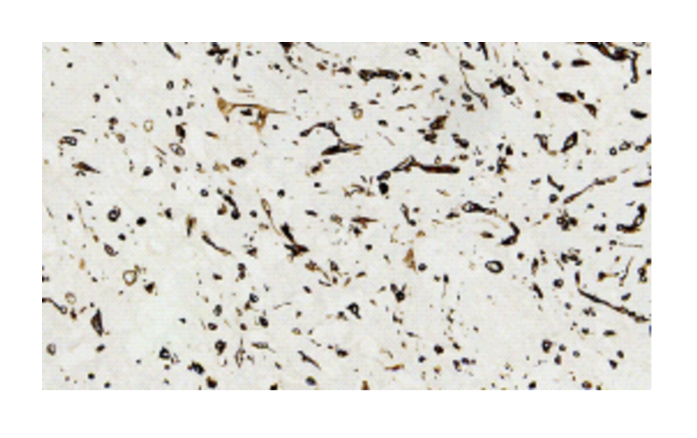

Supplement: Supplementary file 1 — Additional file 1: Fig. S1. Identification of C28/I2 cells was performed through type II collagen staining assay. [file 13018_2023_4238_MOESM1_ESM.tif]

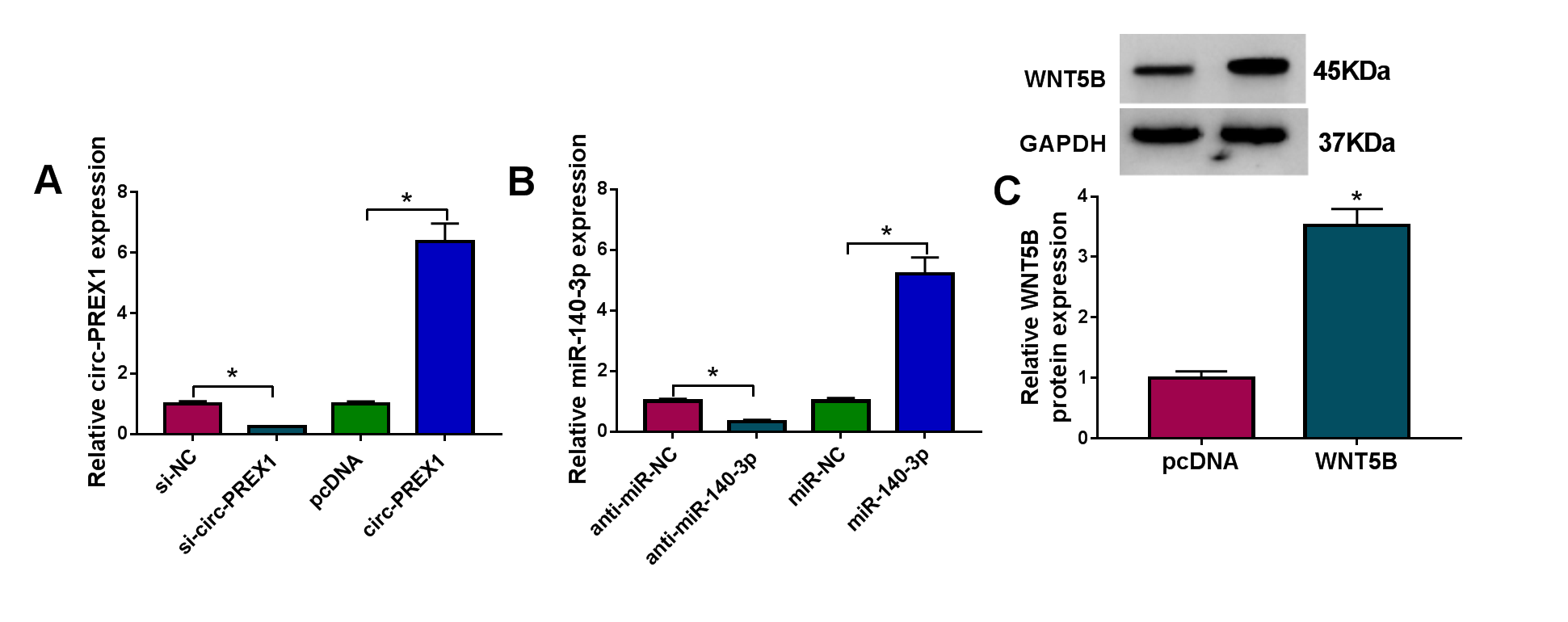

Supplement: Supplementary file 2 — Additional file 2: Fig. S2. Analysis of circ-PREX1, miR-140-3p and WNT5B expression. (A) The effects of siRNA of circ-PREX1 and circ-PREX1 overexpression plasmid on circ-PREX1 expression were analyzed by RT-qPCR. (B) The effects of miR-140-3p mimic and inhibitor on miR-140-3p expression were determined by RT-qPCR. (C) The effect of WNT5B overexpression plasmid on WNT5B expression was determined by western blotting. *P < 0.05. [file 13018_2023_4238_MOESM2_ESM.tif]
